# Trend and Determinants of Mortality Among Women of Reproductive Age: A Twelve-Year Open Cohort Study in Eastern Ethiopia

**DOI:** 10.3389/fgwh.2021.762984

**Published:** 2021-12-14

**Authors:** Merga Dheresa, Abera Kenay Tura, Gamachis Daraje, Mesfin Abebe, Tariku Dingeta, Hirbo Shore, Yadeta Dessie, Tesfaye Assebe Yadeta

**Affiliations:** ^1^School of Nursing and Midwifery, College of Health and Medical Sciences, Haramaya University, Harar, Ethiopia; ^2^Kersa Health and Demographic Surveillance System, College of Health and Medical Sciences, Haramaya University, Harar, Ethiopia; ^3^Department of Obstetrics and Gynecology, University Medical Centre Groningen, University of Groningen, Groningen, Netherlands; ^4^Department of Statistics, College of Computing and Informatics, Haramaya University, Harar, Ethiopia; ^5^School of Public Health, College of Health and Medical Sciences, Haramaya University, Harar, Ethiopia

**Keywords:** women of reproductive age, mortality, Ethiopia, KHDSS, verbal autopsy

## Abstract

**Background:** With only less than a decade left till 2030, it is essential to research the burden and trends of women of reproductive age (WRA) mortality in order to design appropriate interventions toward achieving goal three of the sustainable development goals (SDGs), good health and well-being. For several low-income countries, such data are often lacking or sometimes extrapolated from non-representative facility-based studies. In this paper, we presented trends, causes, and determinants of mortality among reproductive-age women under follow-up for 12 years through the Health and Demographic Surveillance System (HDSS) located in eastern Ethiopia.

**Methods:** We used 12 years of (2008 to 2019) open cohort data of women aged 15–49 living in Kersa HDSS in Eastern Ethiopia. In the HDSS, data on socio-demographic and basic household conditions are recorded for every household member at enrollment, and data on vital events such as births, deaths, and migration were collected and updated biannually as the event happened. Mortality was determined by automated verbal autopsy (InterVA) algorism. We assessed trends in women's reproductive age mortality and the associated determinants using crude and adjusted Cox regression models.

**Results:** In the 12-years cohort, we followed 74,790 women of reproductive age for 339,909.26 person-years-at-risk of observation (PYO), of whom 919 died. Overall, the standardized mortality rate was 270 per 100,000 PYO. There was a notable increase in mortality in the first 3 years (2009 to 2011) which then declined significantly (*p* = 0.0001) until 2019. Most of the deaths were caused by HIV/AIDS (27.88%) and pulmonary tuberculosis (10.62%). In the adjusted Cox regression analysis, the hazard of death was higher among rural residents (AHR, 2.03: 95% CI: 1.60–2.58), unemployed women (AHR, 1.50: 95% CI: 1.19–1.89), and women with no formal education (AHR, 1.24: 95% CI: 1.01–1.52).

**Conclusion:** The study showed a high number of women of reproductive age are still dying mainly due to causes for which preventable strategies are known and have been successfully implemented. The study identified that the main causes of death were related to HIV/AIDS and tuberculosis, and there was a higher hazard of mortality among rural residents, unemployed women, and those with no formal education, who need effective implementation in achieving the SDG three.

## Introduction

Women of reproductive age in low-income countries are the most vulnerable population with the highest risk of dying (1). The fact that women are disproportionately poor, are low in their social status, and have reproductive roles means they are exposed to high health risks (2). There is a significant discrepancy in WRA mortality over the world (1, 3–5). Human immune virus (HIV), tuberculosis (TB) (6), malaria, pregnancy-related infection, hemorrhages (7), complications of pregnancy (8), cerebrovascular accidents, and breast cancer (9) have been reported to attribute to WRA mortality.

Several low-income countries do not have a strong system to capture WRA deaths to design tailored interventions through accounting for the mortality. Having accurate statistics on women's health is essential for formulating and implementing the right mix of policies and programs to meet their health needs (10). The national representative community-based evidence in low-income countries is needed to design appropriate strategic plans and effective intervention (11). In many sub-Saharan countries, the national representative community-based surveys (DHS) study did not report the causes of mortality of disaggregated women of reproductive age (11, 12). Similarly in Ethiopia, where there is low health-seeking behavior and the majority of deaths occur at home, DHS reports lack specific data on the cause and determinants of women of reproductive age mortality (13). As such, health and demographic surveillance systems (HDSS) were established to fill this gap.

A health demographic surveillance system uses verbal autopsy for assigning causes of deaths through the use of a group of physicians or algorithms after collecting data in the community about the deaths from closest relative(s) (14). To minimize human error and synchronize the process, the use of automated verbal autopsy (InterVA) is introduced (15). The method provides sufficient evidence to guide public health priorities in communities in which physician certifications of death are largely unavailable (16). In Ethiopia, where civil registration and vital statistics are underutilized, data related to women of reproductive age mortality was obtained from health facilities and cross-sectional studies. The cross-sectional and health-facility-based study poorly estimates mortality rates and trends representing the community. There is limited research in Ethiopia that estimate the mortality, trends, and its determinants of women of reproductive age mortality.

In addition, since the majority of women's reproductive health deaths happen in the community, the health facility data do not show the true situation of women's reproductive health mortality estimate and do not suggest generalizing the result to other sites. The Health and Demographic Surveillance System obtains data through women's reproductive age observation on an open cohort of all individuals permanently living in specific geographical boundaries. High-quality, trustworthy, and real-time, population data can be obtained through HDSS, reliable continuous and longitudinal data, which helps the government to measure the progress of WRA mortality reduction toward the Sustainable development goals (SDG) (17). Thus, for the specific locations within which they operate, HDSS data will be important for measuring progress in WRA mortality against the SDGs. Therefore, we presented our findings on trends, causes, and determinants of reproductive age mortality in a population-based study in the Kersa Health and Demographic Surveillance System cohort in eastern Ethiopia from 2008 to 2019. Reliable evidence is very important for evidence-based priority setting, planning, and implementation.

## Methods

### Study Setting and Period

The Kersa Health Demographic Surveillance System (KHDSS) field site is located in the eastern part of Ethiopia and was established in 2007 to serve as a health research center for Haramaya University College of Health and Medical Sciences. The initial census began within 12 kebeles (small administrative unit in Ethiopia) to define the baseline denominator population (18). In Ethiopia, a Keble (small administrative unit) has 3,000–5,000 individuals. In rural areas each Keble has one health post lead by 1–3 female health extension workers to provide primary health care for the community. In an urban setting, the population in one Keble is much higher than in a rural setting. Moreover, urban Kebeles have better access to high-level health facilities. After baseline census, the HDSS includes continuous longitudinal recordings of demographic data to monitor the population at regular intervals, observing relevant changes that occur in the designated population. KHDSS field site was extended to the Harari Region (Harar town) in 2012 and operates among six kebeles. Thereafter, in 2015 each HDSS site (Kersa and Harar town) expanded their coverage and doubled the number of kebeles being monitored. Currently, the KHDSS covers 36 kebeles (24 kebeles from Kersa districts and 12 kebeles from Harar town) with 41,056 households, covering a total population of 197,268 (19).

### Study Design and Population

Kersa HDSS is an open dynamic cohort study design linked through time. All individuals living in Keras HDSS were followed. All households living within a specific geographical boundary were visited twice a year; at each visit, changes in the vital status of all household members, births, deaths, migration, household assets, and employment/education status are recorded and updated. Death is one of the events that is regularly updated during each visit. All women of reproductive age living in the study area were our study population.

### Sample Size and Sampling Techniques

All women of reproductive age living in the study area from January 1, 2008, through December 31, 2019, were included.

### Data Collection Procedure

Data were collected using an electronic device tablet computer with an Open Data Kit (ODK) collection application by well-trained regular HDSS staff through face-to-face interviews. Field supervisors were assigned to supervise the data collection process in the field and checked data quality using GPS coordinates, consistency, and validity of the response before it was sent to the database. If supervisors found a data quality problem, they sent it back to the data collectors for correction. The collected data were temporarily stored on ODK aggregate. Then, after checking the quality of the data, data managers migrated the data from temporary storage to the final Open HDS database. To minimize recall bias, the family members of the deceased women were approached after 45 days of mourning, not delayed beyond 6 months.

### Measurement and Variables

Researchers for this study extracted 12 years of data stored from January 1, 2008–December 31, 2019, from the Kersa HDSS open database. Women of reproductive age both alive and dead were retrieved by years. Women of reproductive age mortality was calculated per 100,000 person-years-at-risk of observation (PYO) for each year. During the period, a total of 74,790 women of reproductive age were registered in the database. The data for the causes of deaths were collected using the WHO standard verbal autopsy questionnaire (16). A verbal autopsy questionnaire consists of signs and symptoms of a disease. The cause of the death was determined based on a computerized verbal autopsy algorism. A verbal autopsy algorism involves collecting signs and symptoms during death using a verbal autopsy questionnaire by well-trained data collectors; the data was sent to the database software. The cause of death was determined by a group of physicians or currently determined by automated algorism using InterVA software. InterVA software assigned the probability of cause-specific mortality fraction among the deceased. A group of physicians with different specialties and experiences in VA analysis were involved. InterVA-4 software delivers causes of death compatible with the International *Classification of Diseases version 10* (ICD-10) (16, 20, 21). Based on InterVA-4, the top ten causes of death among reproductive-age women were determined. All independent variables were linked to the women of reproductive age through location and individual identifiers.

Time to events is the period from the start of observation (15 years) until the occurrence of the event (death). The death referred to the study subject who had experienced the interest of event (had died) during the observation period. In this study, censored refers to the study subject who had not experienced death during the follow-up period. Women's reproductive age death within 15 to 49 years of life was reported by close relatives responding to the VA interview.

The wealth index was used to distinguish relatively “rich” and “poor” households. Since our study participants were both from urban (Harar town) and rural areas (Kersa), the assets used to measure wealth index vary for both dwellers. So, the wealth index for urban (Harar town) and rural (Kersa) was calculated separately and appended at the end. Before analysis, categorical variables were changed into binary as yes (present, coded as “1”) or no (absent, coded as “0”). The continuous variables were kept as scale but not string. Variables with the frequency of <5% or >95% are not helpful to differentiate the wealth index in the group, thus we excluded them from the analysis. The wealth index was computed by using principal component analysis (PCA) based on items assessing household assets and possessions. The score of the first component or factor comprising several heavily loaded variables and accounting for the largest variation in the data was categorized into quintiles where each individual falls into a poor, middle, or rich wealth index.

### Statistical Analysis

STATA version 14 (StataCorp 2015, College Station, TX) statistical software was utilized for data analysis. Before analysis, data were cleaned and edited. The reproductive mortality rate was described in each year with 95% CI. The association of predictors with women's reproductive age mortality was assessed with Cox proportional regression model. A Bi-variable Cox regression model was fitted for each explanatory variable. An explanatory variable at bivariate analysis with a *p*-value < 0.2 were included in the multivariable Cox regression model. The Hazard Ratio (HR) and 95% Confidence Intervals (95% CI) were calculated for each variable. The results were reported in an adjusted Hazard ratio (AHR) with 95% confidence interval. Multi-collinearity was assessed using variance inflation factors (VIF). Finally, variables with *P*-value < 0.05 in the multivariable regression were considered as significant predictors of mortality.

### Ethical Clearance

Kersa HDSS has obtained ethical clearance from the national ethical review committee of the Science and Technology Minster of Ethiopia and from the Institutional Health Research Ethics Review Committee (IHRERC) of College of Health and Medical Sciences, Haramaya University, Ethiopia.

## Results

### Socio Demographic Characteristics

In the 12-year period, a total of 74,790 reproductive-age women were followed, resulting in 339909.26 person-years of observation. Three-fourths (74.8%) of them entered into the system through enumeration while the rest were by immigration, 60% reside in rural areas, and 44.93% had no formal education (Table 1).

**Table 1:** Socio-demographic characteristics of all reproductive age and deceased women under the Kersa Health Demographic Surveillance System, Eastern Ethiopia from 2008 to 2019.

**Variables**	**Number**	**Percent**
**Residence area**		
Urban	29,918	40.00
Rural	44,872	60.00
**Ethnicity (*****n*** **=** **74,276)**		
Oromo	53,439	71.95
Amhara	13,830	18.62
Somali	357	0.48
Gurage	2,712	3.65
Harari	3,071	4.13
Tigrawa	428	0.58
Other	439	0.59
**Religion (*****n*** **=** **74,311)**		
Muslim	55,299	74.42
Orthodox	16,559	22.28
Protestant	2,109	2.84
Catholic	222	0.30
Others	122	0.16
**Educational status**		
Literate	39,896	53.34
Read and write	1,290	1.72
No formal education	33,604	44.93
**Occupational status (*****n*** **=** **74,290)**		
Housewife	34,204	46.04
Daily laborer	2,705	3.64
Merchant	5,237	7.05
Student	16,696	22.47
Unemployed	10,707	14.41
Paid employee	4,741	6.38
**Wealth Index (*****n*** **=** **64,657)**		
Poor	20,287	31.38
Middle	21,388	33.08
Rich	22982	35.54

### Women Reproductive Age Mortality

During the total of 339909.26 person-years-at-risk of observation (PYO), 919 reproductive age women died, corresponding with the standardized mortality rate of 270 per 100,000 PYO. Over the follow-up period, the mortality rate did not show any trends until 2012 where the trend started to decrease until 2018 (Table 2). Among the deceased women of reproductive age, 912 of them had a recorded marital status. Five hundred thirty-two (58%) of them were married, 141 (15.4%) were widowed/divorced, and 239 (26.2%) of them were single.

**Table 2:** Women reproductive age mortality in Kersa HDSS, Eastern Ethiopia from 2008 to 2019.

**Years**	**Person-year**	**Death**	**Mortality rate/10,0000**	**95% CI**
2008	11144.13	50	448.67	340.05–591.97
2009	11505.01	43	373.75	277.19–503.95
2010	12066.17	50	414.38	314.07–546.74
2011	13087.66	61	466.09	362.65–599.04
2012	16267.34	51	313.51	238.27–412.52
2013	23435.84	67	285.89	225.01–363.23
2014	24105.45	71	294.54	233.41–371.67
2015	35801.35	90	251.39	204.47–309.08
2016	47244.39	107	226.48	187.39-273.73
2017	47482.73	106	223.24	184.54–270.05
2018	48332.62	102	211.04	173.81–256.24
2019	49436.58	121	244.76	204.81–292.49
Overall	339909.26	919	270.37	253.44–288.42

*CI, Confidence Interval*.

### Cause Specific Mortality

**Figure 1:**
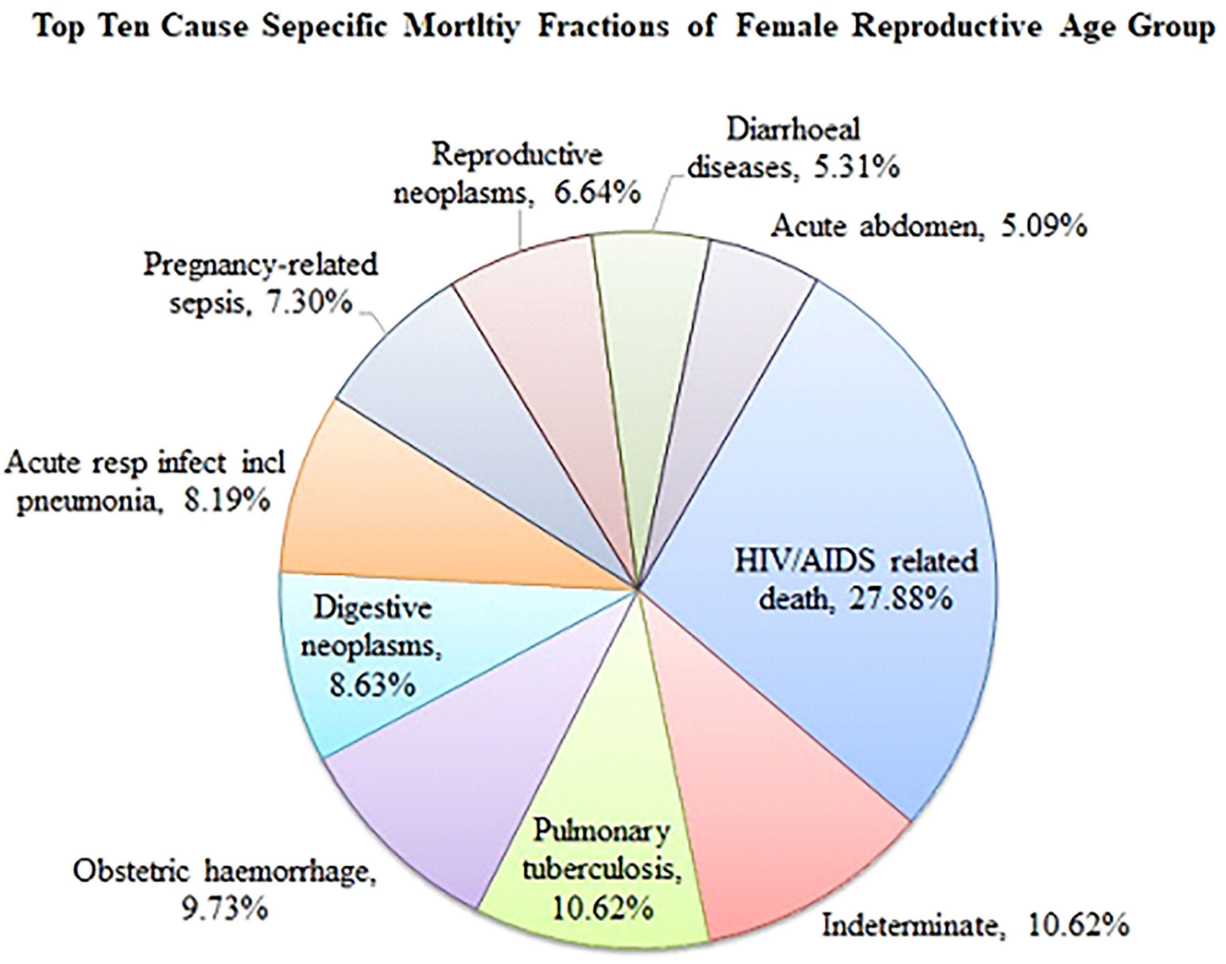
Top 10 cause specific mortality fraction among reproductive age women in Kersa HDSS, eastern Ethiopia from 2008 to 2019.

The top 10 cause-specific mortality fraction (CSMF) revealed that HIV/AIDS-related causes (27.88%) and pulmonary tuberculosis (10.62%) were the leading causes of death (Figure 1).

### Trends of Women Reproductive Age Mortality

**Figure 2:**
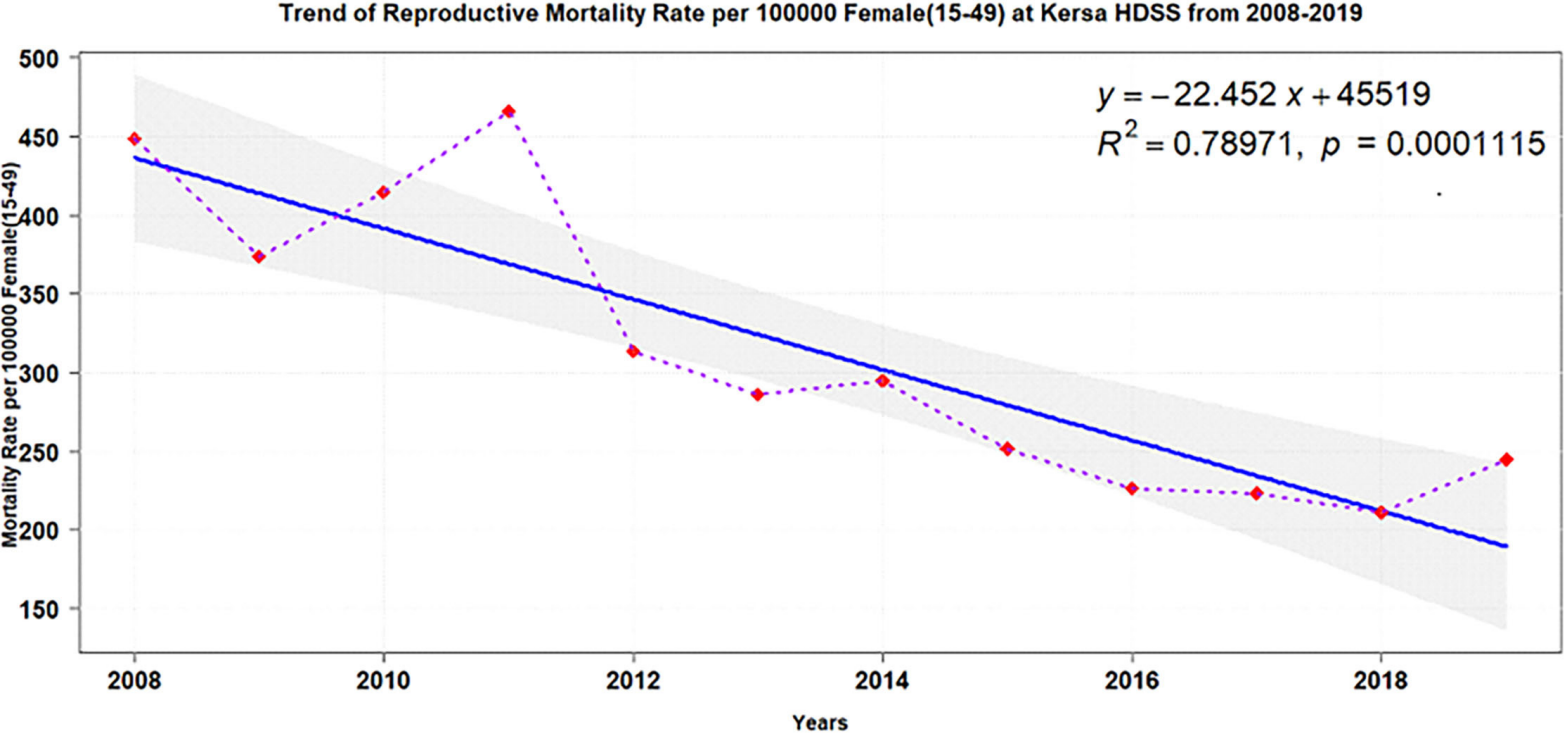
Trends of women reproductive age mortalities in KHDSS, eastern Ethiopia (2008-2019).

Over the 12 years of follow-up, overall mortality declined (adjusted test for trend: *p* = 0.0001) y = −22.452x+45519. Two periods could be distinguished: an increase from 2008 to 2011 followed by a stable continuous decline until 2018 (Figure 2).

### Place of Death

**Figure 3:**
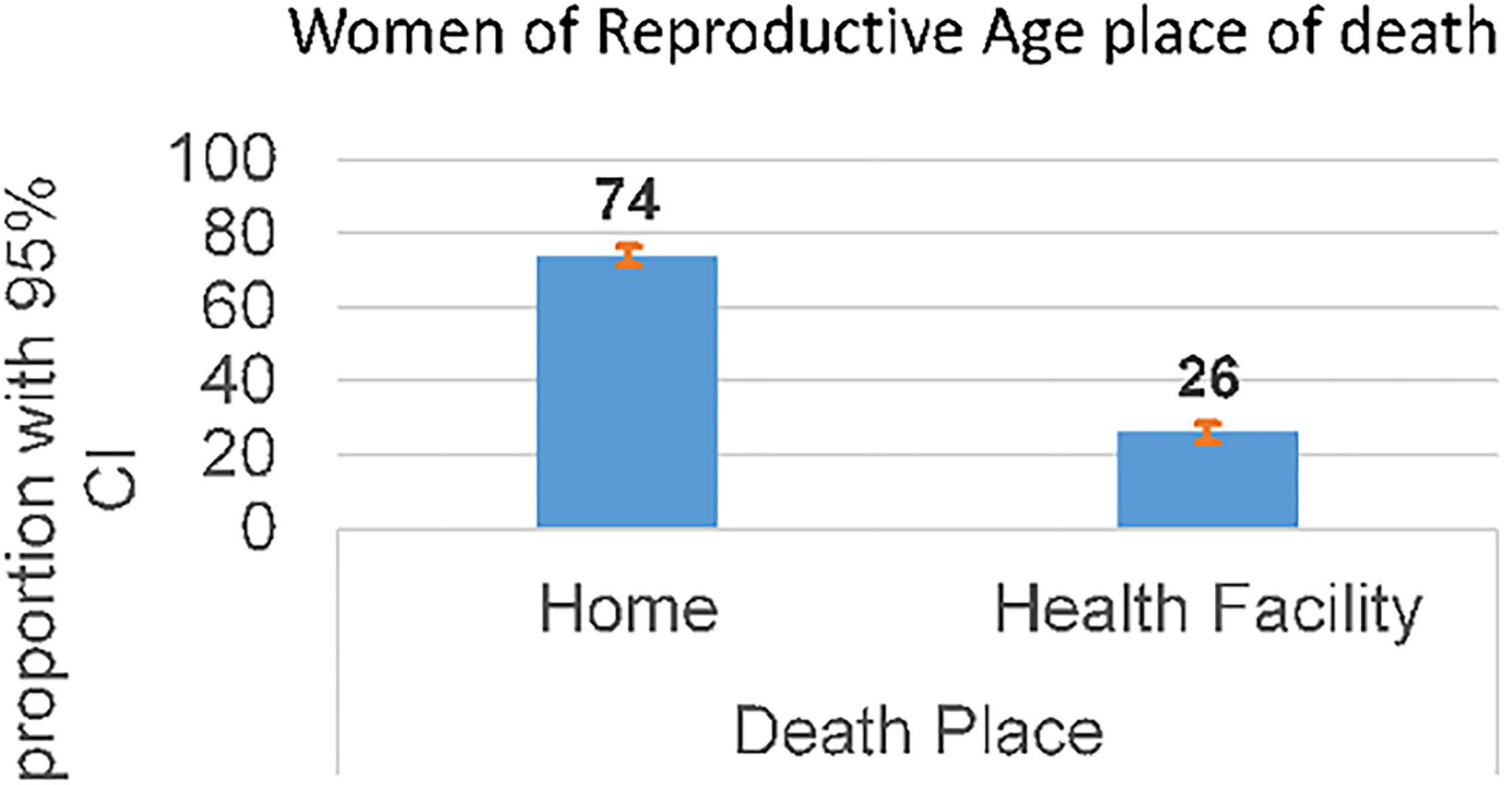
Place of delivery of reproductive age women mortalities in KHDSS, eastern Ethiopia (2008-2019).

The majority of the reproductive age group deaths occurred at home (Figure 3).

### Determinants of Women of Reproductive Age Mortality

In the adjusted cox regression analysis, the hazard of death was higher among rural residents (AHR, 2.03; 95% CI: 1.60–2.58), unemployed women (AHR 1.50; 95% CI: 1.19–1.89), and women with no formal education (AHR 1.24; 95% CI: 1.01–1.52) (Table 3).

**Table 3:** Crude and adjusted Cox regression of hazard of death among women reproductive age in Kersa HDSS, Eastern Ethiopia from 2008 to 2019.

**Variables**	**Status**		**CHR, 95% CI**	**AHR, 95% CI**
	**Alive *n* (%)**	**Died *n* (%)**		
**Residence**				
Urban	29,719 (99.33)	199 (0.67)	1	1
Rural	44,152 (98.40)	720 (1.60)	2.47, 2.11–2.90	2.03, 1.60–2.58
**Occupation**				
House wife	33,644 (98.36)	560 (1.64)	1	1
Daily laborer	2,674 (98.85)	31 (1.15)	0.75, 0.52–1.08	1.13, 0.72–1.75
Merchant	5,187 (99.05)	50 (0.95)	0.61, 0.46–0.82^*^	1.15, 0.81–1.64
Student	16,625 (99.57)	71 (0.43)	0.45, 0.34–0.60^*^	0.68, 0.49–0.94
Unemployed	10,549 (98.52)	158 (1.48)	1.40, 1.14–1.72^*^	1.50, 1.19–1.89^*^
Paid employee	4,694 (99.01)	47 (0.99)	0.59, 0.44–0.80^*^	1.06, 0.71–1.57
**Educational status**				
Literate	39,592 (99.24)	304 (0.76)	1	1
Can read and write	1,269 (98.37)	21 (1.63)	2.05, 1.32–3.19^*^	1.54, 0.96–2.48
Neither read nor write	3,3010 (98.23)	594 (1.77)	2.03, 1.76–2.34^*^	1.24, 1.01–1.52^*^
**Wealth Index**				
Poor	20,030 (98.73)	257 (1.27)	1	1
Middle	21,104 (98.67)	284 (1.33)	1.04, 0.88–1.23	1.15, 0.92–1.29
Rich	22,723 (98.87)	259 (1.13)	0.88, 0.75–1.10	0.99, 0.84–1.19

* *Significantly associated; n, number; CHR, Crude Hazard Ratio; AHR, Adjusted Hazard Ratio; CI, confidence interval*.

## Discussion

In this study, we presented 12 years of trends and causes of women reproductive age mortality in an open cohort in eastern Ethiopia. The overall standard mortality rate during the study period was 270 per 10,0000 PYO. Unstable trends of mortality, in the beginning, was followed by a steady rise from 2009 to 2011 before it started to decline significantly. HIV/AIDS-related causes and tuberculosis were the leading causes of mortality. We found that the hazards of deaths were higher among rural residents, unemployed women, and those with no formal education.

Despite the fact that the trends of WRA mortality in this study were significantly reduced, the mortality is still inadmissibly high. Similarly, high WRA mortality was reported in sub-Saharan Africa (3, 4). In this study, about 70 percent of deaths occurred due to HIV/AIDS (28.8%), pulmonary tuberculosis (10.6%), obstetric hemorrhage (9.3%), acute respiratory infection (8.19%), pregnancy-related sepsis (7.30%), and diarrheal disease (5.31%). For theses causes of deaths, preventable strategies are known and can be successfully implemented with locally affordable resources and technology. In addition, in Ethiopia there is low and delayed health-seeking behavior (13); about 69.9% of WRA perceived barriers of health care access (22), non-adherence to medication, and high cost for health care utilization, which might be contributors to death (13). In this study, a majority (74%) of the deaths occurred at home; this suggests low utilization of health care among WRA. There is a need to establish mechanisms for successful health promotion, prevention, and health care utilization specific to the target groups at large in the community. The health extension workers, which enabled significant improvements in communicable diseases, hygiene and sanitation, knowledge, and health care seeking to be achieved in Ethiopia, need to support the WRA in networking with the community and health facilities (23). Kersa HDSS site should also collaborate with the local health administrators and frontline health care workers in advancing the progress in the reduction of WRA mortality toward the SDGs till 2030 and beyond (3).

Although the HIV prevalence rate in Ethiopia decreased from 2005 to 2011, it remained unchanged between 2011 and 2016. In rural areas, where about 85% of Ethiopians live, the epidemic is also on the rise. Unfortunately, the HIV test uptake among the WRA group was 20% in 2011 (24), and only one quarter (25.2%) of Ethiopian WRA had a comprehensive knowledge of HIV/AIDS (25). The factors associated with HIV-related death among WRA might be due to low health seeking behavior, low highly active antiretroviral therapy coverage, and low adherence. In addition, women in low income countries are more likely to be victims of discrimination in economic, social, and political life, sexual violence, and harmful traditional practices which increases their chance of acquiring HIV (4). Moreover, women are biologically more vulnerable due to infected semen remains in the vaginal canal, exposed mucosal surface area, and vagina susceptible to small tears being factors for HIV infections (26, 27).

Congruent with other studies, we found that reproductive-age women who reside in rural areas have an increased risk of mortality (4). It is challenging for rural residents to access healthcare facilities (28); this may be due to the inaccessibility of health facilities or lack of transportation. A higher WRA mortality that occurs in the home shown in this study supports this claim.

In this study, the hazard of WRA mortality was high among women with no formal education. Education as a factor in mortality decline was reported elsewhere (24, 29, 30). Increasing levels of educational attainment are likely to improve the capability of women to obtain, process, and understand basic health information, including reproductive health services needed to make appropriate health decisions. Moreover, educated women may be less likely to accept traditional explanations for life and death and instead take on broad information about their health, all of which are of key importance in the drive to reduce mortality. Furthermore, more educated women are likely to be more confident about asking questions about their health care needs and are more likely to be listened to by health care professionals (29).

Unemployment was associated with WRA mortality in this study. A similar finding was reported in Georgia (5). The causal mechanisms are related to the rewards that are involved with paid work: financial security, social contacts, a sense of meaning, structured daily activities, as well as psychosocial rewards such as self-esteem or prestige (31, 32). Consequently, job loss has negative consequences as well. The lack of financial security (33, 34) might affect the use of health care and is also a continuous source of stress. Unemployed people are also more likely to have lower psychological well-being and higher levels of stress (35), which may eventually lead to a deterioration of physical health. Moreover, a higher likelihood of risk behavior, such as tobacco or alcohol consumption, has been observed among the unemployed (35, 36).

No significant associations were observed between the women of reproductive age mortality and wealth index. The wealth index might have taken an indirect effect, which was not considered in this study. Assets are central to a vigorous understanding of wealth and poverty, but how to accurately record the relationship and its changes over time is a full of difficulty (5, 37). The asset index may not be linear over time (38). This indicates the need for researchers to further explore the different approaches through which the wealth index could have an effect on WRA mortality.

This study has the following strengths. First, the data were collected from an open cohort over 12 years and therefore it can generate reliable data on the trends of mortality. Second, since the vital events were updated biannually, it may not be subjected to recall bias. However, our study also has some limitations. Since the cause of death was assigned by automated probability, and not by the physician, the cause of death may not be accurate and only a cause with the highest probability was taken, unlike in the group of physicians who might assign some causes. Moreover, not all important predictors of death in women of reproductive age were available in the Kersa HDSS database.

## Conclusion

The study showed a high number of women of reproductive age are still dying mainly due to causes for which preventable strategies are known and have been successfully implemented. The identified main causes of death are related to HIV/AIDS and tuberculosis. A higher hazard of mortality among rural residents, unemployed women, and those with no formal education means they need an effective implementation in achieving the Sustainable Development Goal's 2030 targets and beyond.

## Data Availability Statement

All essential data required for the conclusion are included in the study. The data is a property of Haramaya University and would not be made public due to ethical restrictions. However, additional data may be obtained from the HDSS following the institutional data request and sharing policy.

## Ethics Statement

The studies involving human participants were reviewed and approved by Kersa HDSS has obtained Ethical Clearance from the National Ethical Review Committee and from Haramaya University College of Health and Medical Science and the Institutional Health Research Ethical Review Committee (IHRERC). The patients/participants provided their written informed consent to participate in this study.

## Author Contributions

All authors contributed to the acquisition, analysis and interpretation of data, took part in writing the article, revising it critically for important intellectual content, final approval to be published, and agree to be accountable for all aspects of the work.

## Funding

Hararghe HDSS was funded partly by CDC and Haramaya University. The funders have no role in the design, collection, analysis, or interpretation of findings or the decision to publish. We never received any funds for publication.

## Conflict of Interest

The authors declare that the research was conducted in the absence of any commercial or financial relationships that could be construed as a potential conflict of interest.

## Publisher's Note

All claims expressed in this article are solely those of the authors and do not necessarily represent those of their affiliated organizations, or those of the publisher, the editors and the reviewers. Any product that may be evaluated in this article, or claim that may be made by its manufacturer, is not guaranteed or endorsed by the publisher.
